# Maximum Correntropy Unscented Kalman Filter for Spacecraft Relative State Estimation

**DOI:** 10.3390/s16091530

**Published:** 2016-09-20

**Authors:** Xi Liu, Hua Qu, Jihong Zhao, Pengcheng Yue, Meng Wang

**Affiliations:** 1 School of Electronic and Information Engineering, Xi’an Jiaotong University, Xi’an 710049, China; qh@mail.xjtu.edu.cn (H.Q.); zhaojihong@mail.xjtu.edu.cn (J.Z.); heaven.1452@stu.xjtu.edu.cn (P.Y.); 2 School of Software Engineering, Xi’an Jiaotong University, Xi’an 710049, China; avictor.wally@stu.xjtu.edu.cn

**Keywords:** unscented Kalman filter (UKF), unscented transformation (UT), maximum correntropy criterion (MCC)

## Abstract

A new algorithm called maximum correntropy unscented Kalman filter (MCUKF) is proposed and applied to relative state estimation in space communication networks. As is well known, the unscented Kalman filter (UKF) provides an efficient tool to solve the non-linear state estimate problem. However, the UKF usually plays well in Gaussian noises. Its performance may deteriorate substantially in the presence of non-Gaussian noises, especially when the measurements are disturbed by some heavy-tailed impulsive noises. By making use of the maximum correntropy criterion (MCC), the proposed algorithm can enhance the robustness of UKF against impulsive noises. In the MCUKF, the unscented transformation (UT) is applied to obtain a predicted state estimation and covariance matrix, and a nonlinear regression method with the MCC cost is then used to reformulate the measurement information. Finally, the UT is adopted to the measurement equation to obtain the filter state and covariance matrix. Illustrative examples demonstrate the superior performance of the new algorithm.

## 1. Introduction

The relative state estimation problem is rather important in space communication networks, including inter-satellite link (ISL) establishment, communication support, multiple spacecraft formation flying, and spacecraft rendezvous and docking [1,2,3,4]. Many relative motion scenes make use of the radar or/and ladar to obtain the relative measurement information among spacecrafts, which is necessary to determine the relative motions.

As for the state estimation problem, the Kalman filter (KF) is often used for a linear dynamic system, which is, in essence, a recursive minimum $l_{2}$-norm linear filter [5,6,7]. However, from [8], we know that the relative state estimation in a circular reference orbit involves a linear state Clohessy-Wiltshire (CW) equation and a nonlinear measurement equation. Especially, when the reference orbit is elliptic, the dynamic state equation is also nonlinear [9]. To handle the nonlinear problems, the widely used methods are the extended Kalman filter (EKF) [10,11,12] and unscented Kalman filter (UKF) [11,12,13]. The EKF is a popular nonlinear extension of KF, which makes use of the first order Taylor series expansions to approximate the nonlinear system and applies the KF to this approximation. Nevertheless, the approximation is crude and it may lead to filter divergence if the function exhibits highly non-linear characteristics. Furthermore, the derivation of the Jacobian matrices is cumbersome and often results in the implementation difficulties. In UKF, the probability distribution of the state is approximated by a set of deterministically selected sigma points and propagated though the non-linear process and measurement equations. UKF does not need to calculate the cumbersome Jacobian matrices and provides derivative-free higher-order approximations to achieve more accurate performances than the EKF. Unfortunately, the EKF and UKF may fail to perform well when the measurements are disturbed by some heavy-tailed non-Gaussian noises, which arises naturally in many real applications of engineering. The main reason for this is that both methods are based on the minimum $l_{2}$-norm technique and thus exhibit sensitivity to heavy-tailed noises [14]. To address this problem, some robust methods have been proposed. The Huber’s generalized maximum likelihood methodology is very common [15,16], which is a combined minimum $l_{1}$ and $l_{2}$-norm technique.

Besides Huber’s robust statistics, the optimization criteria in information theoretic learning (ITL) [17,18] provide an alternative effective way, using information theoretic quantities estimated directly from the data as the optimization costs instead of using the usual second-order statistical measures (such as variance and covariance). The ITL costs can capture higher-order statistics of data, and can be used as a robust adaptation cost to achieve excellent performance in a number of applications [19,20,21,22,23,24,25,26,27,28,29,30,31]. Particularly, an optimization criterion based on correntropy, called the maximum correntropy criterion (MCC) [17,18], has recently been successfully applied in robust adaptive filtering in the presence of heavy-tailed non-Gaussian noises [17,28,29,30,31,32].

In this paper, a non-linear Kalman type filter based on MCC has been derived, namely the maximum correntropy unscented Kalman filter (MCUKF). In MCUKF, the UT is applied to obtain a predicted state estimation and covariance matrix, and a non-linear regression model under MCC is used to reformulate the measurement information. Then, the UT is adopted to the measurement equation to obtain the filter state and covariance matrix. Similar to the original UKF, the new filter also has a recursive structure and is suitable for online implementation. It is worth noting that the proposed MCUKF is different from the algorithm in [33].

The organization of the paper is as follows. In Section 2, we provide a short review of the MCC and the traditional UKF. In Section 3, the MCUKF algorithm is derived. Two illustrative examples are then provided in Section 4 to show the excellent performance of the MCUKF. Finally, Section 5 gives the conclusion.

## 2. Preliminaries

### 2.1. Maximum Correntropy Criterion

Given two random variables X,Y∈R with joint distribution function $F_{XY}\left(x,y\right)$, the correntropy is a generalized similarity measure between *X* and *Y* defined by

(1) $$V\left(X,Y\right)=E\left[\kappa (X,Y)\right]=\int \kappa \left(x,y\right)dF_{XY}\left(x,y\right),$$

where E is the expectation operator, and κ(·,·) denotes a shift-invariant kernel following the Mercer condition. In this paper, the kernel function is chosen as the Gaussian kernel given by

(2) $$\kappa \left(x,y\right)=G_{\sigma }\left(e\right)=\exp\left(-\frac{e^{2}}{2\sigma^{2}}\right),$$

where e=x−y, and σ>0 is the kernel bandwidth.

In many practical applications, we have only a limited amount of data and the joint distribution $F_{XY}$ is unknown. In these cases, the correntropy can be estimated by the sample mean estimator:

(3) $$\hat{V}\left(X,Y\right)=\frac{1}{N}\sum_{i=1}^{N}G_{\sigma }\left(e(i)\right),$$

where e(i)=x(i)−y(i), with ${\left\{x(i),y(i)\right\}}_{i=1}^{N}$ being *N* samples drawn from $F_{XY}$.

Taking the Taylor series expansion of the Gaussian kernel yields

(4) $$V\left(X,Y\right)=\sum_{n=0}^{\infty }\frac{{\left(-1\right)}^{n}}{2^{n}\sigma^{2n}n!}E\left[{\left(X-Y\right)}^{2n}\right].$$

It can be seen that the correntropy is a weighted sum of all even order moments of the error variable X−Y. In addition, the kernel bandwidth appears as a parameter weighting the second order and higher order moments. It is noted that the correntropy will be dominated by the second order moment when the kernel bandwidth is very large compared to the dynamic range of the data.

Given a sequence of error data ${\left\{e(i)\right\}}_{i=1}^{N}$, the cost function of MCC is given by

(5) $$J_{MCC}=\frac{1}{N}\sum_{i=1}^{N}G_{\sigma }\left(e(i)\right).$$

Assume W is a parameter vector of an adaptive system to learn, and let x(i) and y(i) denote the model output and the desired response, respectively. The MCC based learning can be formulated as the following optimization problem:

(6) $$\hat{W}=\underset{W\in \Omega }{\arg\max}\frac{1}{N}\sum_{i=1}^{N}G_{\sigma }\left(e(i)\right),$$

where $\hat{W}$ denotes the optimal solution, and Ω denotes a feasible set of the parameter.

### 2.2. Unscented Kalman Filter

The UKF provides a suitable alternative for EKF to deal with nonlinear systems. In this paper, we discuss a discrete-time nonlinear system described as

(7) $$x\left(k\right)=f\left(k-1,x(k-1)\right)+q\left(k-1\right),$$

(8) $$y\left(k\right)=h\left(k,x(k)\right)+r\left(k\right),$$

where $x\left(k\right)\in R^{n}$ denotes the *n*-dimensional state vector of the system at time step *k*, $y\left(k\right)\in R^{m}$ denotes an *m*-dimensional measurement vector. f represents a nonlinear system function, and h is a nonlinear measurement function and they are assumed to be continuously differentiable. The process noise q(k−1) and measurement noise r(k) are mutually uncorrelated, with zero mean values and covariance matrices

(9) $$E\left[q\left(k-1\right)q^{T}\left(k-1\right)\right]=Q\left(k-1\right),\;E\left[r\left(k\right)r^{T}\left(k\right)\right]=R\left(k\right).$$

In general, similar to KF, the UKF includes two steps, namely predict and update:

#### 2.2.1. Predict

First, a set of 2n+1 sigma points are computed by the estimated state and covariance matrix of last time step:

(10) $$\begin{array}{cc} \chi^{0}\left(k-1|k-1\right)=\; & \hat{x}\left(k-1|k-1\right),\\ \chi^{i}\left(k-1|k-1\right)=\; & \hat{x}\left(k-1|k-1\right)\\ \; & +{\left(\sqrt{\left(n+\lambda )P(k-1|k-1\right)}\right)}_{i},i=1,\ldots ,n,\\ \chi^{i}\left(k-1|k-1\right)=\; & \hat{x}\left(k-1|k-1\right)\\ \; & -{\left(\sqrt{\left(n+\lambda )P(k-1|k-1\right)}\right)}_{i-n},i=n+1,\ldots ,2n, \end{array}$$

where ${\left(\sqrt{\left(n+\lambda )P(k-1|k-1\right)}\right)}_{i}$ is the *i*-th column of the matrix square root of (n+λ)P(k−1|k−1), *n* denotes the dimension of the state vector, and *λ* represents a compound scaling factor defined by

(11) $$\lambda =\alpha^{2}\left(n+\varepsilon \right)-n,$$

where *α* determines the extent of the spread of the sigma points, which is usually chosen as 0<α≤1, *ε* is a scaling factor and is usually set to 3−n.

The transformed set is given through the process model

(12) $${\chi^{i}}^{*}\left(k|k-1\right)=f\left(k-1,\chi^{i}\left(k-1|k-1\right)\right),i=0,\ldots ,2n.$$

Then, the predicted state mean and associated covariance matrix are calculated by

(13) $$\hat{x}\left(k|k-1\right)=\sum_{i=0}^{2n}w_{m}^{i}{\chi^{i}}^{*}\left(k|k-1\right),$$

(14) $$\begin{array}{cc} P(k|k-1)= & \sum_{i=0}^{2n}w_{c}^{i}\left[{\chi^{i}}^{*}\left(k|k-1\right)-\hat{x}\left(k|k-1\right)\right]\\ & \times {\left[{\chi^{i}}^{*}\left(k|k-1\right)-\hat{x}\left(k|k-1\right)\right]}^{T}+Q\left(k-1\right), \end{array}$$

where the corresponding weights of the state and covariance matrix are defined as

(15) $$\begin{array}{c} w_{m}^{0}=\frac{\lambda }{\left(n+\lambda \right)},\\ w_{c}^{0}=\lambda /\left(n+\lambda \right)+\left(1-\alpha^{2}+\beta \right),\\ w_{m}^{i}=w_{c}^{i}=\frac{1}{2(n+\lambda )},i=1,\ldots ,2n. \end{array}$$

with *β* being related to prior knowledge of the distribution of x(k) (for Gaussian noises, β=2 is optimal).

#### 2.2.2. Update

Similarly, a set of 2n+1 sigma points are computed by the predicted state mean and covariance matrix

(16) $$\begin{array}{cc} \chi^{0}\left(k|k-1\right)=\; & \hat{x}\left(k|k-1\right),\\ \chi^{i}\left(k|k-1\right)=\; & \hat{x}\left(k|k-1\right)\\ \;+ & {\left(\sqrt{\left(n+\lambda )P(k|k-1\right)}\right)}_{i},i=1,\ldots ,n,\\ \chi^{i}\left(k|k-1\right)=\; & \hat{x}\left(k|k-1\right)\\ \;- & {\left(\sqrt{\left(n+\lambda )P(k|k-1\right)}\right)}_{i-n},i=n+1,\ldots ,2n. \end{array}$$

The transformed set is given through the measurement model

(17) $$\gamma^{i}\left(k\right)=h\left(k,\chi^{i}\left(k|k-1\right)\right),i=0,\ldots ,2n.$$

Then, the predicted measurement mean and covariance matrix are calculated by

(18) $$\hat{y}\left(k\right)=\sum_{i=0}^{2n}w_{m}^{i}\gamma^{i}\left(k\right),$$

(19) $$P_{\mathrm{yy}}=\sum_{i=0}^{2n}w_{c}^{i}\left[\gamma^{i}\left(k\right)-\hat{y}\left(k\right)\right]{\left[\gamma^{i}\left(k\right)-\hat{y}\left(k\right)\right]}^{T}+R\left(k\right).$$

In addition, the state-measurement cross-covariance matrix is computed by

(20) $$P_{\mathrm{xy}}\left(k\right)=\sum_{i=0}^{2n}w_{c}^{i}\left[\chi^{i}\left(k|k-1\right)-\hat{x}\left(k|k-1\right)\right]{\left[\gamma^{i}\left(k\right)-\hat{y}\left(k\right)\right]}^{T}.$$

Next, the Kalman gain is given as

(21) $$K\left(k\right)=P_{\mathrm{xy}}P_{\mathrm{yy}}^{-1}.$$

Finally, the filter state in this case is obtained by

(22) $$\hat{x}\left(k|k\right)=\hat{x}\left(k|k-1\right)+K\left(k\right)\left(y\left(k\right)-\hat{y}\left(k\right)\right).$$

Additionally, the filter covariance is recursively updated as

(23) $$P\left(k|k\right)=P\left(k|k-1\right)-K\left(k\right)P_{\mathrm{yy}}K^{T}\left(k\right).$$

## 3. Unscented Kalman Filter under MCC

To improve the robustness of the UKF, we present the idea to combine the MCC cost function with the UKF framework to derive a novel UKF, which may perform much better in non-Gaussian noise environments, since correntropy contains second and higher order moments of the error.

First, we consider a nonlinear model given by Equation (7) and Equation (8), combine expression Equations (13) and (14) and recast the nonlinear regression model as

(24) $$\left[\begin{array}{c} \hat{x}\left(k|k-1\right)\\ y(k) \end{array}\right]=\left[\begin{array}{c} x(k)\\ h(k,x(k)) \end{array}\right]+\psi \left(k\right),$$

where ψ(k) denotes by

(25) $$\psi \left(k\right)=\left[\begin{array}{c} \eta (x(k))\\ r(k) \end{array}\right],$$

with

(26) $$\begin{array}{cc} & \Psi (k)\\ =\; & E\left[\psi \left(k\right)\psi^{T}\left(k\right)\right]\\ =\; & \left[\begin{array}{cc} P(k|k-1) & 0\\ 0 & R(k) \end{array}\right]\\ =\; & \left[\begin{array}{cc} T_{p}\left(k|k-1\right)T_{p}^{T}\left(k|k-1\right) & 0\\ 0 & T_{r}\left(k\right)T_{r}^{T}\left(k\right) \end{array}\right]\\ =\; & T\left(k\right)T^{T}\left(k\right), \end{array}$$

where T(k) can be obtained by Cholesky decomposition of Ψ(k). Left multiplying both sides of Equation (24) by $T^{-1}\left(k\right)$, we have the following equation

(27) D(k)=g(k,x(k))+e(k),

where $D\left(k\right)=T^{-1}\left(k\right)\left[\begin{array}{c} \hat{x}\left(k|k-1\right)\\ y(k) \end{array}\right]$, $g\left(k,x\left(k\right)\right)=T^{-1}\left(k\right)\left[\begin{array}{c} x(k)\\ h(k,x(k)) \end{array}\right]$, $e\left(k\right)=T^{-1}\left(k\right)\psi \left(k\right)$ and the covariance of e(k) is the identity matrix.

Then, we define the following cost function $J_{L}\left(x(k)\right)$ based MCC:

(28) $$J_{L}\left(x(k)\right)=\sum_{i=1}^{L}G_{\sigma }\left(d_{i}\left(k\right)-g_{i}\left(k,x\left(k\right)\right)\right),$$

where $d_{i}\left(k\right)$ is the *i*-th element of D(k), $g_{i}\left(k,x\left(k\right)\right)$ is the *i*-th row of g(k,x(k)), and L=n+m is the dimension of D(k).

Under the MCC, the optimal estimate of x(k) can be found by maximizing the cost function

(29) $$\hat{x}\left(k\right)=\arg\max_{x(k)}J_{L}\left(x(k)\right)=\arg\max_{x(k)}\sum_{i=1}^{L}G_{\sigma }\left(e_{i}\left(k\right)\right),$$

where $e_{i}\left(k\right)$ is the *i*-th element of e(k) and

(30) $$e_{i}\left(k\right)=d_{i}\left(k\right)-g_{i}\left(k,x(k)\right).$$

Hence, the optimal solution for x(k) can be found from the following equation

(31) $$\frac{\partial J_{L}\left(x(k)\right)}{\partial x(k)}=0,$$

which can also be expressed as

(32) $$\sum_{i=1}^{L}\varphi \left(e_{i}\left(k\right)\right)\frac{\partial e_{i}\left(k\right)}{\partial x_{i}\left(k\right)}=0,$$

when $x_{i}\left(k\right)$ is the *i*-th element of x(k), and $\varphi \left(e_{i}\left(k\right)\right)=G_{\sigma }\left(e_{i}\left(k\right)\right)\cdot e_{i}\left(k\right)$. Then, we define $C\left(k,i\right)=\varphi \left(e_{i}\left(k\right)\right)/e_{i}\left(k\right)$ and have

(33) $$C\left(k,i\right)=G_{\sigma }\left(e_{i}\left(k\right)\right).$$

Equation (32) further writes in matrix form as

(34) $${\left(\frac{\partial g(k,x(k))}{\partial x(k)}\right)}^{T}C\left(k\right)\left[D(k)-g(k,x(k))\right]=0,$$

where

$$C\left(k\right)=\left[\begin{array}{cc} C_{x}\left(k\right) & 0\\ 0 & C_{y}\left(k\right) \end{array}\right],$$

with

$$C_{x}\left(k\right)=diag\left(G_{\sigma }\left(e_{1}\left(k\right)\right),...,G_{\sigma }\left(e_{n}\left(k\right)\right)\right),C_{y}\left(k\right)=diag\left(G_{\sigma }\left(e_{n+1}\left(k\right)\right),...,G_{n+m}\left(e_{n+m}\left(k\right)\right)\right).$$

Based on Equation (27) with only one iteration in [32], we could obtain similar results in the UKF framework by using C(k) to reformulate the measurement information. Similar to [16], there are two ways to do it. One is to re-weight the residual error covariance depending on the value of $e_{i}\left(k\right)=d_{i}\left(k\right)-g_{i}\left(k,x(k)\right)$, the other is to reconstruct the ’pseudo-observation’. In general, the derived results based on two ways are the same. In this paper, we just describe the algorithm based on the first way for simplicity.

Defining $\tilde{\Psi }\left(k\right)$ is the modified covariance, and could be given as

(35) $$\tilde{\Psi }\left(k\right)=T\left(k\right)C^{-1}\left(k\right)T^{T}\left(k\right).$$

For our next discussion, we write $\tilde{\Psi }\left(k\right)$ into two portions so that

(36) $$\tilde{\Psi }\left(k\right)=\left[\begin{array}{cc} {\tilde{\Psi }}_{x}\left(k\right) & 0\\ 0 & {\tilde{\Psi }}_{y}\left(k\right) \end{array}\right].$$

In fact, since the true state x(k) is unknown, we set $\eta \left(x\left(k\right)\right)=\hat{x}\left(k|k-1\right)-x\left(k\right)=0$. In this case, we can see easily that

(37) $$\begin{array}{cc} {\tilde{\Psi }}_{x}\left(k\right)= & T_{p}\left(k|k-1\right)*I*T_{p}^{T}\left(k|k-1\right)\\ = & P(k|k-1). \end{array}$$

The modified measurement covariance can be obtained

(38) $$\tilde{R}\left(k\right)={\tilde{\Psi }}_{y}\left(k\right).$$

In the above derivation, the MCUKF algorithm can be summarized as follows:

1. Choose a proper kernel bandwidth *σ*; set an initial estimate $\hat{x}\left(0|0\right)$ and corresponding covariance matrix P(0|0); and let k=1;
2. Use Equations (10) and (12) to calculate the sigma points and the propagated sigma points with respect to function f;
3. Compute predicted estimate $\hat{x}\left(k|k-1\right)$ and covariance matrix P(k|k−1) by Equations (13) and (14), and adopt Cholesky decomposition to get $T_{p}\left(k|k-1\right)$;
4. Use Equations (16) and (17) to calculate the sigma points and the propagated sigma points with respect to function h;
5. Use Equations (24)–(38) to derive the modified $\tilde{R}\left(k\right)$; compute the predicted measurement mean by Equation (18); and replace R(k) with $\tilde{R}\left(k\right)$ in Equation (19) and have the following covariance matrix:
  (39) $$P_{\mathrm{yy}}=\sum_{i=0}^{2n}w_{c}^{i}\left[\gamma^{i}\left(k\right)-\hat{y}\left(k\right)\right]{\left[\gamma^{i}\left(k\right)-\hat{y}\left(k\right)\right]}^{T}+\tilde{R}\left(k\right).$$
6. Use Equations (20)–(23) to compute the filter state mean and covariance matrix; go back to 2.

**Remark 1.**  *As one can see, different from the UKF algorithm, the MCUKF uses a non-linear regression model combined MCC to update the measurement information. Note that the kernel bandwidth σ is a key parameter in MCUKF. In general, a smaller kernel bandwidth makes the algorithm more robust (with respect to outliers). However, when the kernel bandwidth is too small, it may result in the filter divergence or accuracy deterioration. The reason for this can be seen in [34]. On the other hand, when σ becomes larger and larger, the MCUKF will behave more and more like the UKF algorithm. In particular, the following theorem holds.*

**Theorem 1.**  *If the kernel bandwidth σ approaches infinity, the MCUKF will reduce to the traditional UKF.*

**Proof of Theorem 1.**  The proof is simple, we give a short explanation. Note that as σ→∞, we have C(k)→I. In this case, the MCUKF is equal to the traditional UKF. ☐

Thus, it is seen that the kernel bandwidth has significant influences on the behavior of MCUKF. In practical applications, the kernel bandwidth can be set manually or optimized by trial and error methods.

## 4. Illustrative Examples

This section demonstrates the performances of the proposed algorithm by two illustrative examples. In the paper, we mainly compare the estimation performance based on the following benchmarks:

(40) $${\mathrm{MSE}}_{1}\left(m\right)=\frac{1}{K}\sum_{k=1}^{K}{\left(x\left(k\right)-\hat{x}\left(k|k\right)\right)}^{2},m=1,\ldots ,M,$$

(41) $$\mathrm{MSE}=\frac{1}{M}\sum_{m=1}^{M}{\mathrm{MSE}}_{1}\left(m\right),$$

(42) $${\mathrm{MSD}}_{p}\left(k\right)=\frac{1}{M}\sum_{m=1}^{M}\left({\left(x\left(k\right)-\hat{x}\left(k|k\right)\right)}^{2}+{\left(y\left(k\right)-\hat{y}\left(k|k\right)\right)}^{2}+{\left(z\left(k\right)-\hat{z}\left(k|k\right)\right)}^{2}\right),k=1,\ldots ,K,$$

(43) $${\mathrm{MSD}}_{v}\left(k\right)=\frac{1}{M}\sum_{m=1}^{M}\left({\left(\dot{x}\left(k\right)-\hat{\dot{x}}\left(k|k\right)\right)}^{2}+{\left(\dot{y}\left(k\right)-\hat{\dot{y}}\left(k|k\right)\right)}^{2}+{\left(\dot{z}\left(k\right)-\hat{\dot{z}}\left(k|k\right)\right)}^{2}\right),k=1,\ldots ,K,$$

(44) $${\mathrm{TAMSD}}_{p}=\frac{1}{k_{2}-k_{1}}\sum_{k=k_{1}+1}^{k_{2}}{\mathrm{MSD}}_{p}\left(k\right),$$

(45) $${\mathrm{TAMSD}}_{v}=\frac{1}{k_{2}-k_{1}}\sum_{k=k_{1}+1}^{k_{2}}{\mathrm{MSD}}_{v}\left(k\right),$$

where *K* is the entire time steps in every Monte Carlo run and *M* represents the total Monte Carlo runs.

### 4.1. Example 1

Now, a univariate nonstationary growth model (UNGM) that is often used as a benchmark example for nonlinear filtering is considered, whose state and measurement equations are given by

(46) $$x\left(k\right)=\alpha_{1}x\left(k-1\right)+\alpha_{2}\frac{x(k-1)}{1+x{\left(k-1\right)}^{2}}+\alpha_{3}\cos\left(1.2(k-1)\right)+q\left(k-1\right),$$

(47) $$y\left(k\right)=\frac{x{\left(k\right)}^{2}}{20}+r\left(k\right).$$

The parameters are set to $\alpha_{1}=0.5,\alpha_{2}=25,\alpha_{3}=8$.

First, we consider the case in which all the noises are Gaussian

$$\begin{array}{c} q(k-1)∼N(0,1),\\ r(k)∼N(0,1). \end{array}$$

In this example, the parameters are chosen as K=500,M=100. Table 1 illustrates the MSEs of *x*, defined in Equation (41), in Gaussian noise. Since all the noises are Gaussian, the UKF gives the smallest MSE in all filters. Moreover, it is noted that when the kernel bandwidth is small, the MCUKF may result in a worse estimation; in contrast, when the kernel bandwidth becomes larger, its performance will approach that of the UKF. Actually, it has been proved easily that when σ→∞, the MCUKF will reduce to the traditional UKF (see Theorem 1). Therefore, one should choose a larger kernel bandwidth in Gaussian noises.

Second, we change the observation noise into a heavy-tailed non-Gaussian noise, with a mixed-Gaussian distribution

$$\begin{array}{c} q(k-1)∼N(0,1),\\ r(k)∼0.8N(0,1)+0.2N(0,500). \end{array}$$

Table 2 shows the MSEs of *x* in non-Gaussian measurement noise. As one can see, in this case, when kernel bandwidth is too small or too large, the performance of MCUKF will be not good. However, with a proper kernel bandwidth (say σ=2.0), the MCUKF can outperform the UKF, achieving the smallest MSE. Again, when *σ* is very large, MCUKF achieves almost the same performance as the UKF.

### 4.2. Example 2

**Figure 1:**
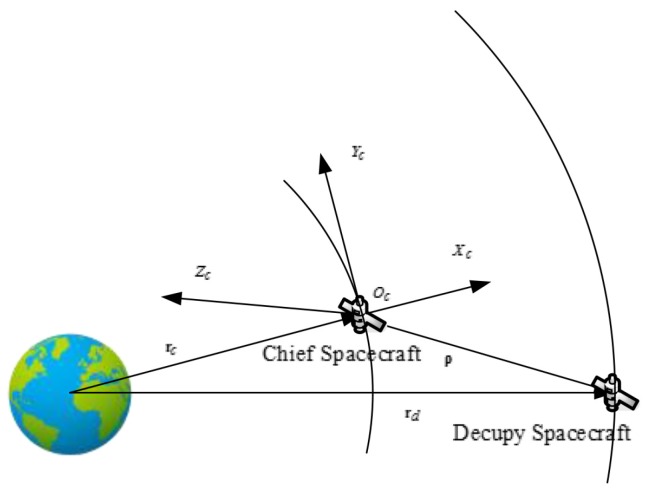
Illustration of example 2.

Finally, we consider a practical example with respect to the relative motion of two spacecrafts, which is illustrated in Figure 1. One of the spacecrafts is called the chief spacecraft, which is moving on the reference orbit, and the other is the deputy spacecraft. They all revolve around the earth and thus the inertial orbital equations of two spacecraft are given as

(48) $${\ddot{r}}_{c}=-\frac{\mu }{r_{c}^{3}}r_{c},$$

(49) $${\ddot{r}}_{d}=-\frac{\mu }{r_{d}^{3}}r_{d},$$

where $r_{c}$ and $r_{d}$ are the position vectors of the chief spacecraft and the deputy spacecraft in ECI coordinate frame, $r_{c}$ and $r_{d}$ are the norms of $r_{c}$ and $r_{d}$, respectively, and *μ* is the gravitational parameter of the earth.

The position vector of the deputy spacecraft relative to the chief spacecraft is

(50) $${æ}_{dc}=r_{d}-r_{c}.$$

Here, we use the Hill coordinate frame, which is a rectangular, Cartesian, dextral rotating frame centered on the chief spacecraft and refer to it as the local-vertical-local-horizontal (LVLH) frame. Then, the motion of the deputy spacecraft relative to the chief spacecraft in the Hill coordinate frame can be described in [35] as

(51) $$\ddot{x}=2\omega \dot{y}+\dot{\omega }y+\omega^{2}x+\frac{\mu }{r_{c}^{2}}-\frac{\mu (r_{c}+x)}{{\left[{\left(r_{c}+x\right)}^{2}+y^{2}+z^{2}\right]}^{3/2}}+q_{1},$$

(52) $$\ddot{y}=-2\omega \dot{x}-\dot{\omega }x+\omega^{2}y-\frac{\mu y}{{\left[{\left(r_{c}+x\right)}^{2}+y^{2}+z^{2}\right]}^{3/2}}+q_{2},$$

(53) $$\ddot{z}=-\frac{\mu z}{{\left[{\left(r_{c}+x\right)}^{2}+y^{2}+z^{2}\right]}^{3/2}}+q_{3},$$

with

(54) $${\ddot{r}}_{c}=r_{c}\omega^{2}-\frac{\mu }{r_{c}^{2}},$$

(55) $$\dot{\omega }=-\frac{2{\dot{r}}_{c}\omega }{r_{c}},$$

where *x*, *y* and *z* are the radial, in-track and cross-track coordinates of the Hill frame, and *ω* denotes the orbital angular velocity of the chief spacecraft.

**Figure 2:**
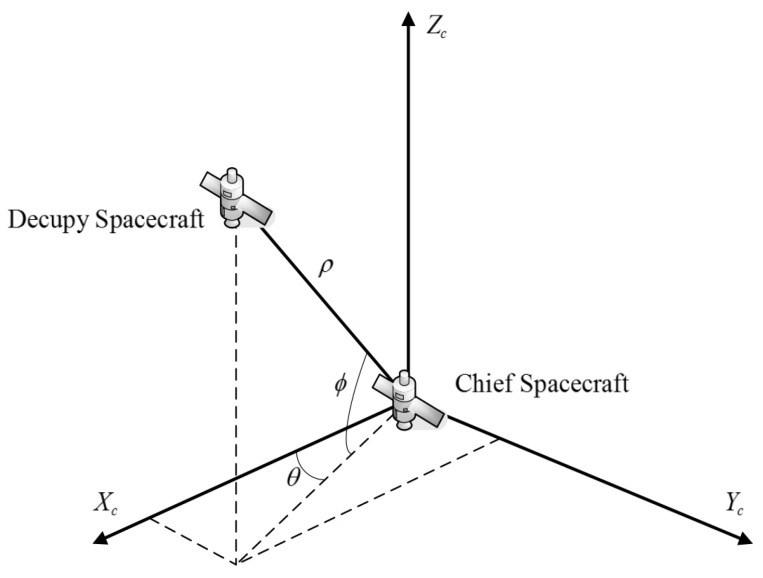
Measurement coordinate system.

The radar is set on the chief spacecraft to obtain the measurements, and the measurement coordinate system is showed in Figure 2. The measurement equations are given as

(56) $$\rho =\sqrt{\left(x^{2}+y^{2}+z^{2}\right)}+r_{1},$$

(57) $$\theta =\tan^{-1}\frac{y}{x}+r_{2},$$

(58) $$\phi =\tan^{-1}\frac{z}{\sqrt{x^{2}+y^{2}}}+r_{3},$$

where *ρ* is the relative range between the chief spacecraft and deputy spacecraft, *θ* is the azimuth angle, and ϕ is the elevation angle.

The two spacecrafts are on the elliptic low Earth orbits. The initial six orbital elements of the chief spacecraft are showed in Table 3, and the trajectory of which in ECI coordinate frame is propagated by the fixed-step fourth-order Runge–Kutta algorithm. The prediction of filters is performed every 0.1 s and the measurements record from the radar every 1 s. The state vector $x\left(k\right)={\left[\begin{array}{cccccc} x(k) & y(k) & z(k) & \dot{x}\left(k\right) & \dot{y}\left(k\right) & \dot{z}\left(k\right) \end{array}\right]}^{T}$ contains the relative position and velocity components in Hill frame, respectively. The initial true state is

$$\begin{array}{c} x\left(0\right)=31.9262\;\mathrm{km},\;\dot{x}\left(0\right)=-0.005583\;\mathrm{km}/s,\\ y\left(0\right)=-7.1384\;\mathrm{km},\;\dot{y}\left(0\right)=-0.071774\;\mathrm{km}/s,\\ z\left(0\right)=33.4729\;\mathrm{km},\;\dot{z}\left(0\right)=0.026249\;\mathrm{km}/s, \end{array}$$

and the initial estimates and covariance matrix of the states are chosen as

$$\begin{array}{c} \hat{x}\left(0|0\right)=31.9262\;\mathrm{km},\;\hat{\dot{x}}\left(0|0\right)=-0.004416\;\mathrm{km}/s,\\ \hat{y}\left(0|0\right)=-8.1384\;\mathrm{km},\;\hat{\dot{y}}\left(0|0\right)=-0.061774\;\mathrm{km}/s,\\ \hat{z}\left(0|0\right)=32.4729\;\mathrm{km},\;\hat{\dot{z}}\left(0|0\right)=0.036249\;\mathrm{km}/s,\\ P\left(0|0\right)=diag(\left[1,1,1,0.01^{2},0.01^{2},0.01^{2}\right]). \end{array}$$

In this example, 100 independent Monte Carlo runs have been conducted, and, in each case, an elapsed time of 7200 s is considered, that is K=7200,M=100. Since correntropy is a local similarity measure, the MCUKF may converge to the optimal solution slowly, especially when the initial values deviate greatly from the true values. Thus, we use the UKF during the first 100 s to make the process settle down and then switch to the MCUKF to continue. The performance of EKF, Huber-EKF (HEKF) [36], UKF and novel robust UKF (NRUKF) [16] are shown for comparison with that of the proposed algorithm.

First, we consider the case in which all the noises are Gaussian, that is,

$$\begin{array}{c} q_{1}∼N\left(0,{\left(10^{-7}\;\mathrm{km}/s^{2}\right)}^{2}\right),\;r_{1}∼N\left(0,{\left(10^{-3}\;\mathrm{km}\right)}^{2}\right),\\ q_{2}∼N\left(0,{\left(10^{-7}\;\mathrm{km}/s^{2}\right)}^{2}\right),\;r_{2}∼N\left(0,{\left(0.05\pi /180\right)}^{2}\right),\\ q_{3}∼N\left(0,{\left(10^{-7}\;\mathrm{km}/s^{2}\right)}^{2}\right),\;r_{3}∼N\left(0,{\left(0.05\pi /180\right)}^{2}\right). \end{array}$$

**Figure 3:**
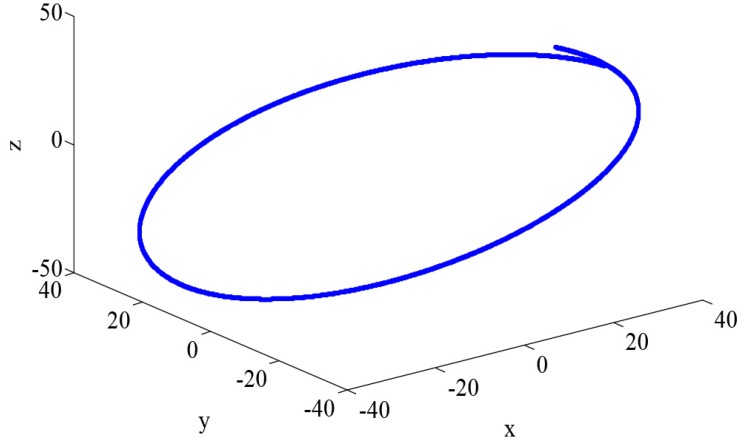
Relative motion of the deputy spacecraft in the Hill frame.

**Figure 4:**
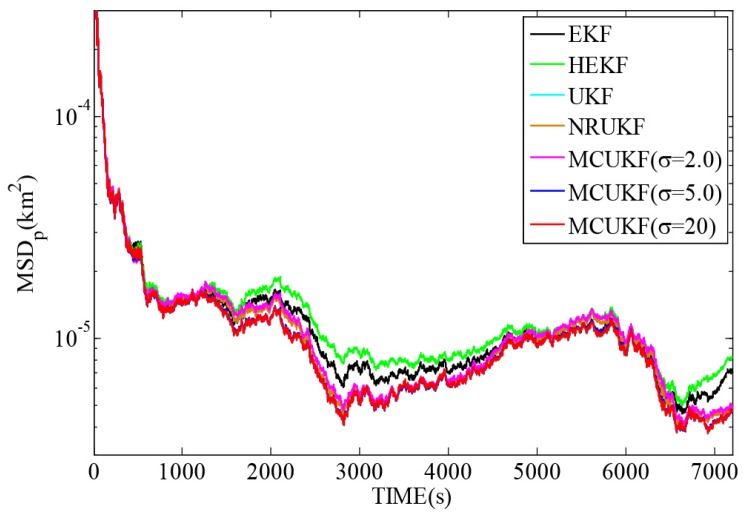
${\mathrm{MSD}}_{p}$ with different filters in Gaussian noises.

**Figure 5:**
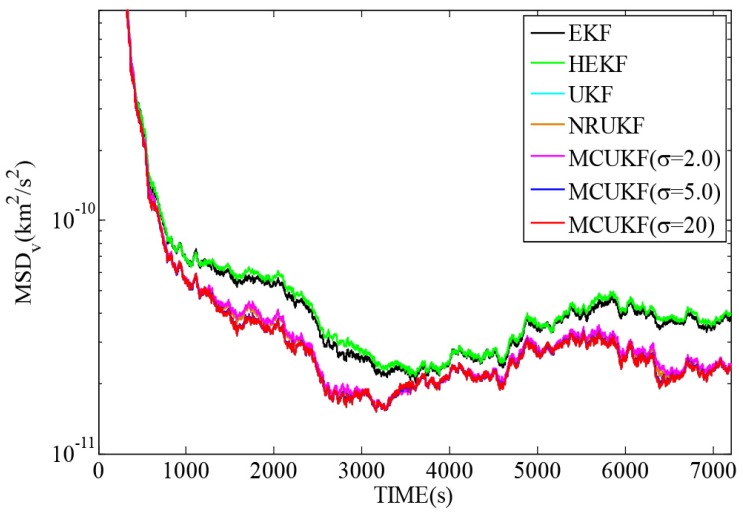
${\mathrm{MSD}}_{v}$ with different filters in Gaussian noises.

Figure 3 describes the relative motion of the deputy spacecraft in the Hill coordinate frame, Figure 4 and Figure 5 demonstrate the ${\mathrm{MSD}}_{p}$ and ${\mathrm{MSD}}_{v}$, defined in Equations (42) and (43), with different filters in Gaussian noises, and Table 4 summarizes the corresponding ${\mathrm{TAMSD}}_{p}$ and ${\mathrm{TAMSD}}_{v}$ , defined in Equations (44) and (45) (the parameter is set to $k_{1}=1000,\;k_{2}=7200$). Those results illustrate that the UKF play the best performance in all filters in this case and the UKF type filters have the better performance than the EKF type counterparts. One can also observe that the robust filters do not perform as well as their non-robust counterparts in the Gaussian noises. Moreover, it is worth noting that when the kernel bandwidth is small, the MCUKF may achieve a worse performance; whereas, when the kernel bandwidth becomes larger, it has similar results to the UKF.

Second, we consider the case in which the measurement noises are heavy-tailed, with mixed-Gaussian distributions

$$\begin{array}{c} q_{1}∼N\left(0,{\left(10^{-7}\;\mathrm{km}/s^{2}\right)}^{2}\right),\\ q_{2}∼N\left(0,{\left(10^{-7}\;\mathrm{km}/s^{2}\right)}^{2}\right),\\ q_{3}∼N\left(0,{\left(10^{-7}\;\mathrm{km}/s^{2}\right)}^{2}\right),\\ r_{1}∼0.9N\left(0,{\left(10^{-3}\;\mathrm{km}\right)}^{2}\right)+0.1N\left(0,{\left(10^{-2}\;\mathrm{km}\right)}^{2}\right),\\ r_{2}∼0.9N\left(0,{\left(0.05\pi /180\right)}^{2}\right)+0.1N\left(0,{\left(0.5\pi /180\right)}^{2}\right),\\ r_{3}∼0.9N\left(0,{\left(0.05\pi /180\right)}^{2}\right)+0.1N\left(0,{\left(0.5\pi /180\right)}^{2}\right). \end{array}$$

**Figure 6:**
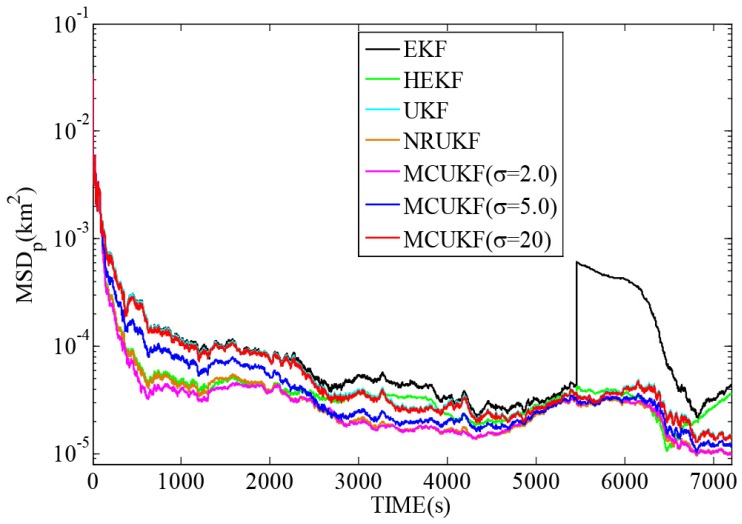
${\mathrm{MSD}}_{p}$ with different filters in non-Gaussian noises.

**Figure 7:**
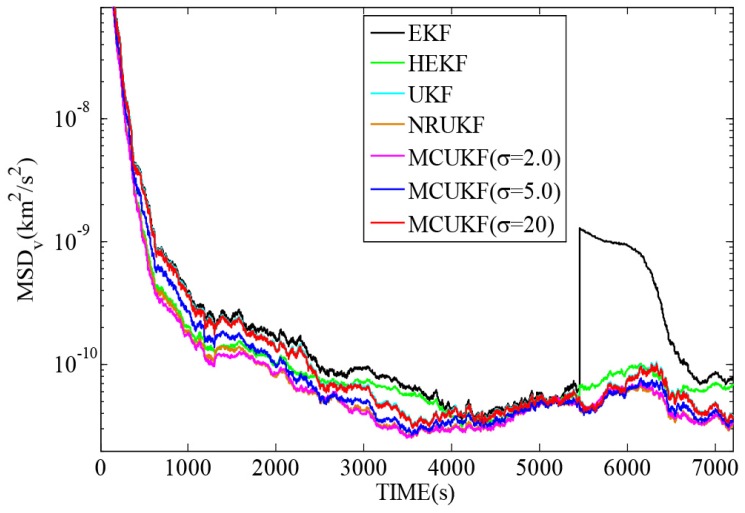
${\mathrm{MSD}}_{v}$ with different filters in non-Gaussian noises.

Figure 6 and Figure 7 reveal the ${\mathrm{MSD}}_{p}$ and ${\mathrm{MSD}}_{v}$ with different filters in non-Gaussian noises, and Table 5 lists the corresponding ${\mathrm{TAMSD}}_{p}$ and ${\mathrm{TAMSD}}_{v}$. As one can observe again, the UKF type filters give a smaller ${\mathrm{TAMSD}}_{p}$ and ${\mathrm{TAMSD}}_{v}$ than the EKF type counterparts. All the robust filters are superior to their non-robust counterparts in impulsive noises. It is noted that when the kernel bandwidth is very large, MCUKF achieves almost the same performance as the UKF. However, with a proper kernel bandwidth, the MCUKF can outperform the UKF significantly. Particularly, when σ=2.0, the MCUKF exhibits the smallest ${\mathrm{TAMSD}}_{p}$ and ${\mathrm{TAMSD}}_{v}$.

Moreover, to compare the computational cost, the computation times of different filters in this example are shown in Table 6. We can see that the computation time of the MCUKF is moderate compared with the UKF, and is superior to that of the HEKF and NRUKF.

## 5. Conclusions

In this paper, we propose a new unscented Kalman filter (UKF), namely the maximum correntropy unscented Kalman filter (MCUKF), which shows strong robustness against heavy-tailed non-Gaussian noises. The proposed algorithm is recast in the form of a non-linear regression model and makes use of the MCC to obtain the filter estimates. The MCUKF is then applied to the spacecraft relative state estimation, compared with EKF, HEKF, UKF and NRUKF. Simulation results confirm that, with a large kernel bandwidth, the MCUKF will behave like the original UKF. With a proper kernel bandwidth, the new filter can achieve better performance than other filters, especially when the measurement system is disturbed by some impulsive non-Gaussian noises.

## Figures and Tables

**Table 1:** MSEs of *x* in Gaussian noises.

Filter	MSE of *x*
UKF	67.6974
MCUKF (σ=2.0)	87.6836
MCUKF (σ=3.0)	80.8406
MCUKF (σ=5.0)	74.0286
MCUKF (σ=10)	72.3362
MCUKF (σ=20)	68.6795

**Table 2:** MSEs of *x* in non-Gaussian measurement noise.

Filter	MSE of *x*
UKF	85.8439
MCUKF (σ=1.0)	84.1944
MCUKF (σ=2.0)	82.6933
MCUKF (σ=3.0)	83.1098
MCUKF (σ=5.0)	84.7173
MCUKF (σ=10)	85.4411

**Table 3:** Initial orbital elements of chief spacecraft.

Orbital Elements	Chief Spacecraft
Semi-major axis	8000 km
Eccentricity	0.150
Orbit inclination	π/6 rad
Argument of perigee	π/6 rad
Right ascension of the ascending node	π/18 rad
True anomaly	0 rad

**Table 4:** ${\mathrm{TAMSD}}_{p}$ and ${\mathrm{TAMSD}}_{v}$ in Gaussian noises.

Filter	${\mathrm{TAMSD}}_{p}$	${\mathrm{TAMSD}}_{v}$
EKF	$9.6397\times 10^{-6}$	$3.7704\times 10^{-11}$
HEKF	$1.0742\times 10^{-5}$	$3.9391\times 10^{-11}$
UKF	$8.4386\times 10^{-6}$	$2.6552\times 10^{-11}$
NRUKF	$9.0277\times 10^{-6}$	$2.7508\times 10^{-11}$
MCUKF $\left(\sigma =2.0\right)$	$9.3516\times 10^{-6}$	$2.8038\times 10^{-11}$
MCUKF $\left(\sigma =5.0\right)$	$8.4909\times 10^{-6}$	$2.6625\times 10^{-11}$
MCUKF $\left(\sigma =20\right)$	$8.4403\times 10^{-6}$	$2.6554\times 10^{-11}$

**Table 5:** ${\mathrm{TAMSD}}_{p}$ and ${\mathrm{TAMSD}}_{v}$ in non-Gaussian noises.

Filter	${\mathrm{TAMSD}}_{p}$	${\mathrm{TAMSD}}_{v}$
EKF	$1.0680\times 10^{-4}$	$2.2751\times 10^{-10}$
HEKF	$3.2302\times 10^{-5}$	$7.5898\times 10^{-11}$
UKF	$4.2884\times 10^{-5}$	$8.4705\times 10^{-11}$
NRUKF	$2.5510\times 10^{-5}$	$5.7120\times 10^{-11}$
MCUKF $\left(\sigma =2.0\right)$	$2.4842\times 10^{-5}$	$5.6557\times 10^{-11}$
MCUKF $\left(\sigma =5.0\right)$	$3.1545\times 10^{-5}$	$6.6535\times 10^{-11}$
MCUKF $\left(\sigma =20\right)$	$4.1800\times 10^{-5}$	$8.3054\times 10^{-11}$

**Table 6:** Computation time comparison.

Filter	Computation Ratio
UKF	1
HEKF	6.81
NRUKF	4.62
MCUKF	3.91
